# Effect of Eye Patching in Rehabilitation of Hemispatial Neglect

**DOI:** 10.3389/fnhum.2013.00527

**Published:** 2013-09-02

**Authors:** Nicola Smania, Cristina Fonte, Alessandro Picelli, Marialuisa Gandolfi, Valentina Varalta

**Affiliations:** ^1^Department of Neurological and Movement Sciences, Neuromotor and Cognitive Rehabilitation Research Center, University of Verona, Verona, Italy

**Keywords:** hemispatial neglect, rehabilitation, perceptual disorders, treatment, stroke, visual stimulation, superior colliculus, eye patching

## Abstract

Eye patching (EP; monocular or right hemifield) has been proposed to improve visuospatial attention to the ignored field in patients with hemispatial neglect. The aim of this paper is to review the literature on the effects of EP in hemispatial neglect after stroke in order to convey evidence-based recommendations to clinicians in stroke rehabilitation. Thirteen intervention studies were selected from the Medline, EMBASE, Scopus, Cochrane Library, CINAHL, PsychINFO, EBRSR, and Health Star databases. Methodological quality was defined according to the Physiotherapy Evidence Database. Overall, seven studies used monocular EP, five used right hemifield patching, and one compared right monocular with right hemifield patching. Seven studies compared normal viewing to monocular or hemifield patching conditions. Six studies included a period of treatment. As to the monocular EP, four studies reported positive effects of right monocular patching. One study showed an improvement in hemispatial neglect with left monocular patching. Two studies found no superiority of right vs. left monocular patching. One study found no effects of right monocular patching. As to the right hemifield EP, one study showed improvements in neglect after right hemifield patching. Three studies found that right hemifield patching combined with another rehabilitation technique was more effective than that treatment alone. One study found no differences between right hemifield patching combined with another treatment and that treatment alone. One study found the same effect between right hemifield patching alone and another rehabilitation technique. Our results globally tend to support the usefulness of right hemifield EP in clinical practice. In order to define a level of evidence with the standard rehabilitation evidence rating tools, further properly powered randomized controlled trials or meta-analysis are needed.

## Introduction

Hemispatial neglect is a common syndrome after stroke in which patients fail to report or respond or be aware of stimuli located contralateral to a brain lesion (Heilman and Valenstein, 1979; Kwon et al., 2012). The incidence of hemispatial neglect varies between 8 and 95% in individuals with stroke (Bowen et al., 1999), with a reasonable estimate of 23% (Pedersen et al., 1997). These epidemiological discrepancies are thought to result from inconsistencies in defining hemispatial neglect, differences in the timing of examination after stroke, the use of different tests to detect visual hemispatial neglect, and the use of small and insensitive test batteries in the available literature (Ogden, 1985; Stone et al., 1991).

Lesions involving the right inferior frontal gyrus, precentral gyrus, postcentral gyrus, superior temporal gyrus, middle temporal gyrus, middle occipital gyrus, insula, and surrounding white matter are those most frequently associated with hemispatial neglect (Chechlacz et al., 2012; Yue et al., 2012).

As left hemispatial neglect (after right brain damage) is the most frequent case in clinical practice, we will refer to this condition throughout the whole paper.

Testing of hemispatial neglect shows that patients misbisect lines to the right of true center, fail to cancel targets on the left side of a page, and fail to draw the left side of objects and scenes (Kwon et al., 2012). Diagnosis must exclude that these behavioral abnormalities arise from a primary sensory or motor deficit such as hemianopia or paralysis (Heilman and Valenstein, 1979).

An accurate estimate of the rates of hemispatial neglect recovery after stroke could not be derived to date (Bowen et al., 1999). However, a recent cohort study on a sample of 101 stroke patients described progress of time as an independent covariate that reflects neurological recovery of hemispatial neglect (Nijboer et al., 2013). The authors found that at 12 weeks after stroke, 54% of the initial hemispatial neglect patients recover from their impairment, and approximately 60% after 26 up to 52 weeks from the onset of stroke (Nijboer et al., 2013). Consequently, in clinical practice it is not unusual to have cases of chronic hemispatial neglect more than 1 year after stroke.

The presence of hemispatial neglect increases postural control abnormalities in patients with stroke. Indeed, they usually show trunk misalignment (van Nes et al., 2009), postural instability (Pérennou et al., 2000), and increased risk of falls (Paolucci et al., 2001; Jutai et al., 2003; Mackintosh et al., 2006). Hemispatial neglect is a recognized predictor of poor functional outcome, with a lower level of independence in activities of daily living (e.g., dressing, bathing, eating, and mobility), prolonged hospital stay, greater need of care-giver support (Katz et al., 1999; Cherney et al., 2001; Buxbaum et al., 2004; Franceschini et al., 2010), and a higher risk of functional deterioration at 1 year post-stroke (Paolucci et al., 2001). Thus, it is not surprising that over the past 60 years more than 18 different rehabilitation techniques have been put forward to alleviate, reduce, or remediate unilateral hemispatial neglect (Luauté et al., 2006; Ogourtsova et al., 2010). The most recent Cochrane review of cognitive rehabilitation for hemispatial neglect after stroke (Bowen and Lincoln, 2007) reports that although several types of neglect-specific approaches can improve performance on some, but not all, standardized neglect tests, evidence to support, or refute their effectiveness in reducing disability and improving independence is still insufficient.

Eye patching (EP) is an interesting approach to hemispatial neglect rehabilitation that has been proposed since the early 1990s as a method to improve visual-scanning and attention toward the neglected field (Butter and Kirsch, 1992). From a clinical point of view, EP may have remarkable gains over other treatment methods because of its high feasibility and low cost. However, the literature about EP reports non-unique evidences of effectiveness. Some of these studies display several methodological limitations. Furthermore, confounding factors in this debate are that studies differ in experimental design and that two different types of EP methods have been proposed.

Although some literature reviews dealing with the effects of hemispatial neglect rehabilitation have been published in the last decade (Butter and Kirsch, 1992; Diamond, 2001; Manly, 2002; Pierce and Buxbaum, 2002; Luauté et al., 2006; Bowen and Lincoln, 2007; Ogourtsova et al., 2010), none have been specifically dedicated to the EP approach.

The main aim of this paper is to review the literature on the effects of EP in post-stroke hemispatial neglect in order to convey evidence-based practice recommendations to clinicians in stroke rehabilitation. Furthermore, given the potential role of this approach in clinical practice, we aim at giving indications for guiding future studies in this field of research.

## Rationale of Eye Patching in Hemispatial Neglect

A number of studies on EP technique in post-stroke hemispatial neglect referred to the Sprague Effect theory (see below for details) (Sprague and Meikle, 1965; Sprague, 1966a,b), while others have interpreted their results in light of a different rationale (*Interhemispheric balance theory* and *Visual exploration constraint theory*) (Arai et al., 1997; Beis et al., 1999; Ianes et al., 2012). On this basis, we decided to propose three main theories in support of the potential benefit of EP in the treatment of hemispatial neglect after stroke.

### The Sprague effect theory

The Sprague effect was first described in 1966 by Sprague. In a remarkable series of studies on animal models (cat), Sprague showed that visually guided behavior is subserved by interactions involving the midbrain and cortical pathways (Sprague and Meikle, 1965; Sprague, 1966a). Sprague reported that hemianopia resulting from a contralateral, large posterior cortical lesion could be partially alleviated by ablation of the superior colliculus contralateral to the cortical lesion or transection of the commissure of the superior colliculus. He observed that cats with contralesional orienting deficits improved their ability to detect stimuli in the contralateral field after surgical ablation of the contralesional superior colliculus. Sprague’s hypothesis that ablation of the contralateral superior colliculus disinhibited the ipsilesional colliculus and improved orientation of contralesional attention (Sprague, 1966b), met with some skepticism and the neural basis for this phenomenon continues to fire debate between supporters and opponents (Soroker et al., 1994; Walker et al., 1996; Arai et al., 1997; Barrett et al., 2001).

With regard to the use of EP in the treatment of left hemispatial neglect in patients with right brain damage, Posner and Rafal (1987) suggested that inhibiting contralesional (left) collicular activity might lessen orienting deficits. They hypothesized that input to the superior colliculi from the eyes may be predominantly monocular and contralateral and that a right eye patch may sensory deprive the left colliculus (Hubel et al., 1975).

### The interhemispheric balance theory

Beis et al. (1999) suggested that wearing patches over both right half-fields in patients with left hemispatial neglect after right brain damage activates the right hemisphere, leading to an increase in the level of leftward attention. Unlike right monocular EP (which is thought to cause simultaneous activation of both hemispheres), covering both right half-fields should activate only the right hemisphere.

**Figure 1:**
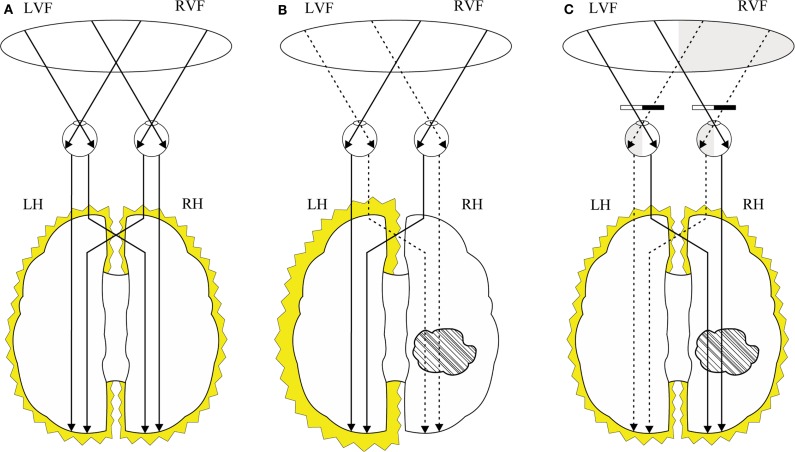
**Interhemispheric balance theory: (A) interhemispheric brain activation in individuals without stroke; (B) interhemispheric imbalance in individuals with right hemisphere stroke where the left hemisphere is activated and right hemisphere is under-activated; (C) patching bilateral right half-fields in individuals with left hemineglect and right hemisphere stroke stimulates the right hemisphere and reduces the stimulation of the left hemisphere leading to the interhemispheric re-balance**. LVF, left visual field; RVF, right visual field; LH, left hemisphere; RH, right hemisphere.

A balance between the hemispheres may be thus established between the “overactivated” damaged right hemisphere and the “non-activated” healthy left hemisphere (Beis et al., 1999) (see Figure 1).

### The visual exploration constraint theory

Some authors (Arai et al., 1997; Ianes et al., 2012) suggest that the use of EP might be viewed as an application of Constraint-Induced Therapy (CIT), a well-known rehabilitation program in patients with upper limb paresis. This treatment aims to reverse the affected limb “learned non-use” phenomenon (Taub et al., 2006). In hemispatial neglect, patients have a strong tendency to orient their exploratory eye movements toward the ipsilesional space. In keeping with a rationale similar to that of CIT in patients with hemispatial neglect, the use of ipsilesional hemifield EP may help patients to visually explore their neglected space (Arai et al., 1997; Ianes et al., 2012).

## Materials and Methods

Original articles were selected from the following electronic databases: Medline (1950–March 2013), EMBASE (1992–March 2013), Scopus (1992–March 2013), the Cochrane Library (2008–March 2013), CINAHL (1992–March 2013), PsychINFO (1992–March 2013), EBRSR (1992–March 2013), and Health Star (1992–March 2013). The following keywords were used: stroke, neglect, visual neglect, unilateral spatial neglect, spatial neglect, hemispatial neglect, attention, eye patching, viewing, patching, glasses neglect, monocular, binocular. Different combinations of all these terms were used to source the articles.

Two independent reviewers (Valentina Varalta, Cristina Fonte) reviewed all abstracts retrieved from the initial search. Studies were included which evaluated the effects of monocular or hemifield EP in patients with hemispatial neglect (intervention studies) as a result of right brain damage. Excluded were non-intervention studies, animal studies, non-English language studies, studies enrolling only healthy subjects, studies involving stroke patients without hemispatial neglect and reviews. The two reviewers selected the relevant articles and performed the quality assessment of the studies. They independently read all the selected articles and listed the details in an appropriate grid (see Table 1). In addition to the electronic search, the reference lists of the selected full-text articles were checked for further articles. Three other investigators (Nicola Smania, Alessandro Picelli, and Marialuisa Gandolfi) read all the relevant articles and provided further assessment of data quality and validity. Disagreements were resolved by discussion. Heterogeneity in the selected studies precluded formal review. Thus, the results presented here are qualitative and represent the views of the investigators.

**Table 1:** **Short description of the studies considered for review**.

Study	Study design	Patients (no.)	Time post onset (days)	Patients with VFD (no.)	Testing procedure	Intervention	Duration of intervention	PEDro score (0–10)
Butter and Kirsch (1992)	Case-series	13	112	8	Line and Letter Cancelation, Reading, Line Bisection, Clock Drawing	Normal viewing right monocular EP	1 Time	NA
		18	29.6	13	Line Bisection	Right monocular EP left visual stimulation right monocular EP + left visual stimulation	1 Time	
Soroker et al. (1994)	Case-series	6	135	3	Line Bisection	Normal viewing right monocular EP left monocular EP	1 Time	NA
Serfaty et al. (1995)	Case-series	26	67.2	12	Star Cancelation	Normal viewing right monocular EP left monocular EP	1 time	NA
Walker et al. (1996)	Case-series	9	506	9	Letter Cancelation, Line Bisection, Clock Drawing, Letter String Reading, Text Reading, Chimeric Face Recognition	Normal viewing right monocular EP left monocular EP	1 Time	NA
Arai et al. (1997)	Case-series	10	255	9	Line Bisection, Line Cancelation, Figures Copying	Normal viewing right hemifield EP	1 Time	NA
Barrett et al. (2001)	Single-case	1	ns	1	Line Bisection	Direct and indirect condition: normal viewing right monocular EP left monocular EP	1 Time	NA
Khurshid et al. (2009)	Single-case	1	365	1	Line Cancelation	Direct and indirect condition: normal viewing right monocular EP left monocular EP	1 Time	NA
Beis et al. (1999)	RCT	22	49.3	ns	Visual-scanning movements: time spent and number of movements, FIM	Group 1: VST + right hemifield EP; Group 2: VST + right monocular EP; Group 3: VST	12 weeks, 12 h/day	2/10
Zeloni et al. (2002)	RCT	11	236.2	9	Line, Letter and Bell Cancelation, Copy of Drawing, Line Bisection	Group 1: VST + right hemifield EP; Group 2: VST	1 week	NA
Fong et al. (2007)	RCT	60	11.9	ex	BIT-c, BIT-b, Clock Drawing, FIM	Group 1: TR + right hemifield EP; Group 2: TR; Group 3: OT	6 weeks, 5 days/week, 1 h/day	6/10
Tsang et al. (2009)	RCT	34	21.8	ns	BIT-c, FIM	Group 1: OT + right hemifield EP; Group 2: OT	4 weeks, 5 days/week, 1 h/day	7/10
Ianes et al. (2012)	RCT	18	12.9	ex	Line and Bell Cancelation, Line Bisection	Group 1: right hemifield EP; Group 2: VST	2 weeks, Group 1: 8 h/day; Group 2: 40 min/day	NA
Wu et al. (2013)	RCT	27	368	ns	CBS, Eye Movements, Trunk-arm Kinematic Analysis	Group 1: CIT + right monocular EP; Group 2: CIT; Group 3: OT	3 weeks, 5 days/week, 2 h/day	7/10

*RCT, randomized controlled trial; VFD, visual field deficit; ns, not specified; BIT-c, behavioral inattention test-conventional subtest; BIT-b, behavioral inattention test-behavioral subtest; FIM, functional independence measure; CBS, Catherine Bergego scale; VST, visual-scanning training; TR, trunk rotation; OT, occupational therapy; CIT, constraint-induced therapy; NA, not applicable*.

Methodological quality of the intervention studies was defined according to the Physiotherapy Evidence Database (PEDro) score as reported in the Physiotherapy Evidence Database (1999). The main author (Nicola Smania) verified all the scores.

## Results

A total of 83 papers were reviewed. Sixty-nine studies were excluded according to the above-mentioned criteria. Thirteen intervention studies were included in the review.

Five were case-series/case-control studies (Butter and Kirsch, 1992; Soroker et al., 1994; Serfaty et al., 1995; Walker et al., 1996; Arai et al., 1997), two were single-case studies (Barrett et al., 2001; Khurshid et al., 2009), and six were randomized controlled trials (RCTs) (Beis et al., 1999; Zeloni et al., 2002; Fong et al., 2007; Tsang et al., 2009; Ianes et al., 2012; Wu et al., 2013).

Seven studies investigated the effects of right monocular EP (five also analyzed the effects of left monocular EP) (Butter and Kirsch, 1992; Soroker et al., 1994; Serfaty et al., 1995; Walker et al., 1996; Barrett et al., 2001; Khurshid et al., 2009; Wu et al., 2013) and five assessed the effects of right hemifield EP (Arai et al., 1997; Zeloni et al., 2002; Fong et al., 2007; Tsang et al., 2009; Ianes et al., 2012). Only one study investigated the effect of right monocular EP and that of right hemifield EP (Beis et al., 1999).

Seven studies compared patient performance on neglect testing under two experimental conditions: normal viewing and viewing during EP (Butter and Kirsch, 1992; Soroker et al., 1994; Serfaty et al., 1995; Walker et al., 1996; Arai et al., 1997; Barrett et al., 2001; Khurshid et al., 2009). Six compared the effects of a rehabilitation technique with the same kind of treatment combined with EP (Beis et al., 1999; Zeloni et al., 2002; Fong et al., 2007; Tsang et al., 2009; Wu et al., 2013) or EP treatment applied alone (Ianes et al., 2012).

Three studies were performed in patients in the early stage after stroke (Fong et al., 2007: mean days = 11.9; Tsang et al., 2009: mean days = 21.8; Ianes et al., 2012: mean days = 12.9), while nine studies were conducted in patients in the sub-acute-chronic phase of illness (Soroker et al., 1994: mean days = 135; Serfaty et al., 1995: mean days = 67.2; Walker et al., 1996: mean days = 506; Arai et al., 1997: mean days = 255; Barrett et al., 2001: not specified; Khurshid et al., 2009: days = 365; Beis et al., 1999: mean days = 49.2; Zeloni et al., 2002: mean days = 236.2; Wu et al., 2013: mean days = 368). One study (Butter and Kirsch, 1992) tested patients at <1 month after the onset of stroke (mean days = 29.6) and patients in the chronic phase (mean days = 112).

The studies are summarized as follows (see also Table 1 for methodological issues):

(1) Butter and Kirsch (1992) conducted two different experiments. In the first one, they tested the performance of 13 stroke patients with hemispatial neglect (co-morbidity: 8 patients with hemianopia; 11 patients with eye movement disturbances; 3 patients with visual extinction) during normal viewing and right monocular EP by means of the following test: Line Cancelation, Letter Cancelation, Reading, Line Bisection, and Clock Drawing. The authors observed that under the EP condition, 11 patients had modest clinical improvement in at least one of the five outcomes, noting statistically significant improvements only in the Line Bisection Test. In their second experiment, Butter and Kirsch tested 18 patients with hemispatial neglect (co-morbidity: 13 patients with hemianopia; 11 patients with eye movement disturbances; 1 patient with visual extinction) by means of a computerized test. Patients were required to bisect a line presented on the video screen at baseline and during presentation of visual warning stimuli on the left end of the line (warning condition). Both these conditions were carried out under normal viewing and under right monocular EP. The authors reported that patients performed significantly better under warning conditions compared to the baseline evaluation. Furthermore, they observed a smaller beneficial effect of right monocular EP compared to presentation of visual warning stimuli on the left end of the line during normal viewing (Butter and Kirsch, 1992).
(2) Soroker et al. (1994) analyzed the severity of hemispatial neglect in six stroke patients (co-morbidity: three patients with hemianopia; three patients with visual extinction) by means of a Line Bisection Test performed under three testing conditions: normal viewing; right monocular EP; and left monocular EP. The authors observed a significant improvement under the right monocular EP condition in one patient. Furthermore, three patients showed a significant worsening under the left monocular EP condition (Soroker et al., 1994).
(3) Serfaty et al. (1995) analyzed 26 stroke patients with hemispatial neglect (co-morbidity: 10 patients with left hemianopia and 2 with left quadrantanopia) by means of the Star Cancelation Test performed under the same conditions used by Soroker et al. (1994). The authors noted a significant improvement during right monocular EP compared to the normal viewing condition in 13 patients. Furthermore, two patients showed non-statistically significant improvements during left monocular EP (Serfaty et al., 1995).
(4) Walker et al. (1996) tested the presence and severity of hemispatial neglect in nine stroke patients (co-morbidity: all patients with left hemianopia) under the same conditions used by Soroker et al. (1994) by means of the following tests: Letter Cancelation, Line Bisection, Letter String Reading, Text Reading, and Chimeric Face Recognition. The authors observed that in the right EP condition three patients improved on at least one test and five patients worsened. In the left EP condition, five patients were found to worsen on at least one test, whereas two patients improved (Walker et al., 1996).
(5) Barrett et al. (2001) examined the effects of monocular EP on perceptual-attention and motor-intentional deficits in one stroke patient with hemispatial neglect (co-morbidity: left lower quadrantanopia) by means of a video Line Bisection Test performed directly (left/right on the video screen corresponded with workspace left/right) and indirectly (a 180°change in camera perspective reversed the image) under three testing conditions: normal viewing; right monocular EP; and left monocular EP. Paradoxically, under the right monocular EP condition, patient perceptual-attention deficit was found to significantly worsen, whereas there was a significant improvement under the left monocular EP condition (Barrett et al., 2001).
(6) Khurshid et al. (2009) analyzed the effects of monocular EP in one stroke patient with hemispatial neglect (co-morbidity: left homonymous hemianopia) by means of the video Line Cancelation Test performed under the same conditions used by Barrett et al. (2001). The authors showed that left monocular EP had no effect, whereas right monocular EP reduced left-sided omissions as compared with the un-patched condition (Khurshid et al., 2009).
(7) Arai et al. (1997) analyzed the performance of 10 stroke patients with hemispatial neglect (co-morbidity: 9 patients with visual field deficits) under normal viewing or during right hemifield EP by means of the following tests: Line Bisection, Line Cancelation, and Figure Copying. The authors found that nine patients showed improvement in hemispatial neglect on at least one of the three tests used during right hemifield EP as compared to the normal viewing condition (it was not specified if improvements were statistically significant). No effects were seen in the other two patients (Arai et al., 1997).
(8) Beis et al. (1999) randomized 22 stroke patients (co-morbidity not specified) into three groups: Group 1 (*n* = 7) received Visual-Scanning Training (VST) plus right hemifield EP; Group 2 (*n* = 7) underwent VST plus right monocular EP; Group 3 (*n* = 8) performed VST alone. All patients underwent 12-week training. They were evaluated before and after treatment by means of the Functional Independence Measure (FIM) and an analytical test recorded by photo-oculography (number of times the subject looked at the left zone; time spent looking at left zone). After treatment, significant improvements were found on the FIM and the number of times the subject looked at the left zone in Group 1 vs. Group 3. No difference was found between Groups 2 and 3. Statistics for within-group comparisons were not reported (Beis et al., 1999).
(9) Zeloni et al. (2002) randomized 11 stroke patients (co-morbidity: 11 patients with left hemiplegia; 9 patients with visual field deficits) into two groups: Group 1 (*n* = 5) received VST plus right hemifield EP; Group 2 (*n* = 6) underwent VST alone. All patients underwent 1-week training. They were evaluated before, immediately after and 1 week post-treatment by means of the following tests: Line Cancelation, Letter Cancelation, Bell Cancelation, Copy of Drawing, and Line Bisection. After treatment, a significant improvement of visual spatial neglect was found in Group 1 vs. Group 2 as measured by the above-mentioned tests. Improvements were maintained at the follow-up evaluation. Within-group comparisons showed significant improvement only in Group 1 at all time points (Zeloni et al., 2002).
(10) Fong et al. (2007) randomized 60 stroke patients (co-morbidity: all patients with left hemiplegia) into three groups: Group 1 (*n* = 20) received voluntary trunk rotation treatment plus right hemifield EP; Group 2 (*n* = 20) underwent voluntary trunk rotation treatment alone; Group 3 (*n* = 20) received occupational therapy. All patients underwent 6-week training. They were evaluated before, immediately after and 1 month post-treatment by means of the Behavioral Inattention Test (BIT), Clock Drawing Test, and FIM. After treatment and at the follow-up evaluation, no significant difference for any outcome measure was found between groups. Statistics for within-group comparisons were not reported (Fong et al., 2007).
(11) Tsang et al. (2009) randomized 34 stroke patients (co-morbidity not specified) into two groups: Group 1 (*n* = 17) performed occupational therapy plus right hemifield EP; Group 2 (*n* = 17) performed occupational therapy alone. All patients underwent 4-week training. They were evaluated before and immediately after treatment by means of the BIT (conventional subtest) and FIM. After treatment, a significant improvement was found in Group 1 vs. Group 2 on the BIT. Within-group comparisons showed significant improvements for all outcome measures in both groups (Tsang et al., 2009).
(12) Ianes et al. (2012) randomized 18 patients (co-morbidity not specified) into two groups: Group 1 (*n* = 10) received right hemifield EP; Group 2 (*n* = 8) underwent VST. All patients underwent 2-week training. They were evaluated before, immediately after and 1 week post-treatment by means of the following tests: Line Cancelation, Bell Cancelation, and Line Bisection. After treatment, no significant difference was found between groups. At the follow-up evaluation, a significant improvement was found in Group 1 vs. Group 2 on the Line Cancelation test. Within-group comparisons showed significant improvements for all outcome measures in both groups (Ianes et al., 2012).
(13) Wu et al. (2013) randomized 27 stroke patients (co-morbidity: all patients with left hemiplegia and 8 patients with visual extinction) into three groups: Group 1 (*n* = 9) received paretic arm CIT plus right monocular EP; Group 2 (*n* = 9) underwent CIT alone; Group 3 (*n* = 9) received occupational therapy. All patients underwent 3-week training. They were evaluated before and immediately after treatment by means of the Catherine Bergego Scale (CBS), Eye Movements (namely: the fixation amplitude from leftmost to rightmost fixation points, the number of fixation points, and the fixation time in the left area), and Arm Kinematic Analysis. In particular, the authors used an eye tracker system to record eye movement by detecting the subject’s pupil during the Line Bisection, as well as a seven-camera motion analysis system to evaluate reaction time, duration of the reaching movement, total distance (the path of the hand in three-dimensional space), planned control of the reaching movement (percentage of movement used for the acceleration phase), and trunk lateral shift to left. After treatment, a significant improvement was found in Group 1 and Group 2 vs. Group 3 for the CBS. Furthermore, a significant improvement was found in Group 2 and Group 3 vs. Group 1 for the left fixation point. As for the Arm Kinematic Analysis, a significant improvement in the pre-planned control of the reaching movements was found in Group 1 vs. Groups 2 and 3 and in trunk lateral shift to left in Group 1 vs. Group 2. Furthermore, a significant improvement in the reaction time was found in Group 2 vs. Group 3. Statistics for within-group comparisons were not reported (Wu et al., 2013).

Overall, seven studies used monocular EP (Butter and Kirsch, 1992; Soroker et al., 1994; Serfaty et al., 1995; Walker et al., 1996; Barrett et al., 2001; Khurshid et al., 2009; Wu et al., 2013), five used right hemifield EP (Arai et al., 1997; Zeloni et al., 2002; Fong et al., 2007; Tsang et al., 2009; Ianes et al., 2012), and one compared the effects of right monocular EP with right hemifield EP (Beis et al., 1999). The duration of intervention, the frequency and the duration of each session varied across studies. Six studies (Beis et al., 1999; Zeloni et al., 2002; Fong et al., 2007; Tsang et al., 2009; Ianes et al., 2012; Wu et al., 2013) compared outcomes before and after a period of treatment, while seven studies compared the performances on neglect tests during normal viewing and wearing monocular (Butter and Kirsch, 1992; Soroker et al., 1994; Serfaty et al., 1995; Walker et al., 1996; Barrett et al., 2001; Khurshid et al., 2009) or hemifield EP (Arai et al., 1997). Only three studies included follow-up evaluations (Zeloni et al., 2002; Fong et al., 2007; Ianes et al., 2012).

As to the monocular EP, four studies reported positive effects of right monocular EP (Butter and Kirsch, 1992; Serfaty et al., 1995; Khurshid et al., 2009; Wu et al., 2013) and one study (Barrett et al., 2001) showed a clear improvement in hemispatial neglect during left monocular EP. Two studies found no clear superiority of right vs. left monocular EP (Soroker et al., 1994; Walker et al., 1996) and one study found no effects of right monocular EP (Beis et al., 1999).

As to hemifield EP, one study showed a clear improvement in hemispatial neglect during right hemifield EP (Arai et al., 1997) and three studies found that the combination of right hemifield EP with another rehabilitation technique was more effective than the same treatment applied alone (Arai et al., 1997; Zeloni et al., 2002; Tsang et al., 2009). One study found no differences between the combination of right hemifield EP with another treatment and the same treatment applied alone (Fong et al., 2007), while one study found the same effect between EP applied alone and another rehabilitation technique (Ianes et al., 2012).

With regard to data interpretation, three studies showed results that were inconsistent with the presence of a *Sprague effect* during monocular EP (Soroker et al., 1994; Walker et al., 1996; Barrett et al., 2001). Indeed, according to Sprague’s collicular hypothesis (Sprague, 1966b), patching the right eye should have decreased the tendency to make eye movements to the right and therefore reduce left hemispatial neglect. However, the results of these three studies showed no clear increase in leftward eye movements after right monocular EP. On the other hand, two studies (Arai et al., 1997; Ianes et al., 2012) suggested that their observations were consistent with the “forced use” intervention (*Visual exploration constraint theory*), and one study suggested that the findings were consistent with the *Interhemispheric balance theory* (Beis et al., 1999).

Finally, seven studies failed to interpret results in light of a specific theory (Butter and Kirsch, 1992; Serfaty et al., 1995; Zeloni et al., 2002; Fong et al., 2007; Khurshid et al., 2009; Tsang et al., 2009; Wu et al., 2013).

## Discussion

The results of the present review showed that EP is a promising procedure in the rehabilitation of patients with hemispatial neglect during the acute, subacute, or chronic phase of stroke. As to the type of EP, the data tend to favor right hemifield EP over monocular EP. The data available to date are insufficient to support or refute the effectiveness of EP at reducing disability and improving patient independence. Few studies investigated maintenance of improvements after EP by short-term follow-up evaluations. The effectiveness of this procedure should be further evaluated by future research.

### Effects of monocular EP

Right monocular EP was the first approach to be examined in patients with hemispatial neglect. Its effects have been tested mostly in case-controls and single-case studies, which reported highly conflicting results. A few studies found that right monocular EP has some effects on improving patient performance during neglect visual search tests (Butter and Kirsch, 1992; Serfaty et al., 1995; Khurshid et al., 2009). Other studies found no clear superiority of right vs. left monocular EP (Soroker et al., 1994; Walker et al., 1996) and one study described unexpected improvement in hemispatial neglect after left monocular EP (Barrett et al., 2001). Only two studies tested the effects of right monocular EP (Beis et al., 1999; Wu et al., 2013) by means of an RCT design. They used specific analytical instruments to test these effects. The earlier study compared the effects of right monocular EP with those of right hemifield EP using photo-oculography and showed that the monocular EP approach was less effective than the right hemifield EP approach in regaining voluntary control over the deficit (Beis et al., 1999). The right hemifield EP indeed increased the number of times the subject looked at the left zone (Beis et al., 1999). This study reached a PEDro score of 2/10, thus indicating that it has some methodological shortcomings. The later study attempted to compare the effects of right monocular EP plus paretic arm CIT with those of CIT or occupational therapy alone. The main outcome was that CIT combined with monocular EP and CIT alone lead to similar beneficial effects on functional performance in patients’ everyday life (Wu et al., 2013). However, these approaches had differential effects on eye movement and reaching kinematics. Indeed, while CIT alone improved eye movements and limb initiation, CIT plus EP facilitated pre-planned control of limb movement, and trunk control (see Results for details). This study reached a PEDro score of 7/10 indicating a fair methodological quality.

Taken together, the studies examining the effect of right monocular EP (Butter and Kirsch, 1992; Soroker et al., 1994; Serfaty et al., 1995; Walker et al., 1996; Beis et al., 1999; Barrett et al., 2001; Khurshid et al., 2009; Wu et al., 2013) on hemispatial neglect are not very convincing; when compared with the right hemifield EP approach, they tend to favor the second technique (Beis et al., 1999). Indeed, the majority were case-control or single-case studies (Butter and Kirsch, 1992; Soroker et al., 1994; Serfaty et al., 1995; Barrett et al., 2001; Khurshid et al., 2009), one RCT had methodological drawbacks (Beis et al., 1999), while another good quality RCT did not display any significant additional effect of monocular EP when combined with CIT (Wu et al., 2013). Moreover, the puzzling evidence that left monocular EP may occasionally lead to an improvement in hemispatial neglect has led some authors to suggest that there is no clear rationale for right monocular EP in hemispatial neglect rehabilitation (Soroker et al., 1994; Walker et al., 1996; Barrett et al., 2001).

### Effects of right hemifield EP

Arai et al. (1997) were the first to examine the effects of right hemifield EP in patients with hemispatial neglect after stroke. In this study, 10 patients with hemispatial neglect were tested under normal viewing or while wearing glasses in which the right portion of the lenses was obscured. During right hemifield EP, 8 out of 10 patients improved their ability to explore the left hemispace (Arai et al., 1997). This study gave new insights into the potential effects of this technique on reducing hemispatial neglect. Following on the study by Arai et al. (1997), five RCTs tested the effects of right hemifield EP in hemispatial neglect (Beis et al., 1999; Zeloni et al., 2002; Fong et al., 2007; Tsang et al., 2009; Ianes et al., 2012). These studies tested the effect of right hemifield EP in conjunction with other rehabilitation procedures (VST, Trunk Rotation, Occupational Therapy, CIT), except for the study by Ianes et al. (2012) that compared the effectiveness of right hemifield EP with a conventional VST for hemispatial neglect (Ianes et al., 2012).

As to methodological quality, three of these RCTs (Beis et al., 1999; Fong et al., 2007; Tsang et al., 2009) were rated by means of the PEDro scale (Physiotherapy Evidence Database, 1999), reaching a score of 2/10, 6/10, and 7/10, respectively. Two other studies (Zeloni et al., 2002; Ianes et al., 2012) could not be rated with the PEDro score because they were not considered as physiotherapy interventions.

Beis et al. (1999), Zeloni et al. (2002), and Tsang et al. (2009) showed that the effect of right hemifield EP in combination with other treatments produced better improvement in hemispatial neglect deficit, than the same treatments applied alone. Only one study compared the effects of right hemifield EP treatment alone against another hemispatial neglect treatment (VST) and found that the right hemifield EP was as effective as conventional neglect treatment (I31). Taking into account that the hemifield EP procedure is far less expensive than VST, which requires one-on-one patient-therapist involvement, the results of this study are very relevant for the clinical practice.

Although the available literature on right hemifield EP is encouraging, some clear methodological limitations of the studies merit attention: small patient sample size (Arai et al., 1997; Beis et al., 1999; Zeloni et al., 2002; Ianes et al., 2012), lack of power, and sample size calculation (Arai et al., 1997; Beis et al., 1999; Zeloni et al., 2002; Ianes et al., 2012), lack of follow-up evaluations (Beis et al., 1999; Tsang et al., 2009), inclusion of patients with visual field deficits (because hemifield patching may be too penalizing in such cases) (Arai et al., 1997; Zeloni et al., 2002), use of unchallenging neglect tests (Arai et al., 1997; Ianes et al., 2012), lack of sample size homogeneity in terms of time from stroke (Arai et al., 1997; Zeloni et al., 2002), and severity of hemispatial neglect (Zeloni et al., 2002). All in all, given the potential of the right hemifield EP approach in remediating hemispatial neglect after stroke, future research with improved methodological quality is warranted.

Another potentially interesting research area is the basis of the effects of right hemifield EP. On the one hand, these effects could be explained by the *Interhemispheric balance theory* according to which right hemifield EP may allow or increase detection and selection of visual inputs from the neglected field. These inputs may enhance activation of the damaged (right) hemisphere, allowing a re-balance between the directional orientation processors of the right and left hemispheres. We may suggest that testing the effects of right hemifield EP in a functional Magnetic Resonance Imaging (MRI) or EEG mapping study in healthy subjects and in patients with hemispatial neglect may help further our understanding of the neural basis of this rehabilitation approach.

On the other hand, right hemifield EP might be viewed as another application of such “forced use” intervention (Arai et al., 1997). Following this conceptual model, use of a right hemifield EP may induce patients to visually explore their neglected space according to the *Visual exploration constraint theory* (Ianes et al., 2012).

### Advantages of EP

Several advantages of EP approaches should be acknowledged. First, it is an inexpensive and easily applicable procedure that requires that patients simply wear spectacles containing monocular or right hemifield EP. It may be used for many hours a day and provide long-term stimulation, a condition not applicable to conventional hemispatial neglect treatments. Second, patients may not be actively involved in one-on-one treatment sessions. This is particularly relevant in patients in whom the clinical condition may interfere with actively participating in treatment sessions due to medical reasons or to a lack of sitting tolerance. Finally, EP approaches may be easily coupled with other rehabilitation techniques or performed at home during daily activities with the support of a caregiver.

All these features make the EP particularly suitable for patients in the acute-sub-acute stage after stroke (Ianes et al., 2012). This last point is especially important because during the first post-stroke period patients may be unable to actively participate in rehabilitation treatment sessions, and could benefit from a treatment regime in which they are passive beneficiaries (Ianes et al., 2012). In addition, trunk misalignment or a lack of trunk postural control in the early stage after stroke may not allow the patient to receive conventional treatment.

### Recommendations to clinicians

Taken together, the results of the present review show that right hemifield EP might be a promising procedure in treating hemispatial neglect. However, providing clear recommendations to clinicians is difficult for several reasons.

First, two RCTs rated 6/10 and 7/10 by the PEDro database displayed partially conflicting results on the effectiveness of right hemifield EP in the early phases after stroke (Fong et al., 2007; Tsang et al., 2009). However, the power of these studies was inadequate because of the small sample size. The authors, who suggested that a replication of the studies with an appropriate patient sample is warranted, admitted this. It is worth noting here that this point highlights a limit of the PEDro scale, in that the presence of an adequate patient sample size is not considered as a criterion for rating methodological quality (Geha et al., 2013).

Second, two RCTs relevant to our review were not found to be eligible for PEDro rating because they were not considered as physiotherapy interventions (Zeloni et al., 2002; Ianes et al., 2012). This precluded the possibility to rate the RCTs by Zeloni et al. (2002) and Ianes et al. (2012) who showed that right hemifield EP combined with another treatment (Zeloni et al., 2002) or applied alone (Ianes et al., 2012) is more effective or at least as effective as a standard VST.

To summarize, the results of the present review globally tend to support the usefulness of right hemifield EP in clinical practice. In order to define a level of evidence by means of the standard rehabilitation evidence rating tools, however, further research is warranted by means of adequately powered RCTs and/or a meta-analysis of the present literature data.

### Directions for future research

Future studies in this field are recommended. These studies should be directed to investigate the effects of EP on reducing hemispatial neglect severity, disability, and to improve patient independence. It is also desirable that the limitations of the current literature are taken into consideration. First, RCTs in large patient samples and with multiple and long-term follow-up evaluation sessions (at least at 1 and 3 months after treatment) are warranted. This is crucial to have reliable evidence about the role of EP in stroke rehabilitation in order to convey a use/not use message to clinicians. Second, studies involving sub-acute patients should be implemented, where spontaneous recovery will need to be considered as a potential confounding factor. The most suitable method to control for the effects of spontaneous recovery would be to include an untreated group. However, the inclusion in the study of an untreated group is difficult to justify, because withholding treatment for hemispatial neglect from a patient is unethical. Instead, a specific study design such as “delayed treatment” should be applied (Paolucci et al., 2000). Third, patients with hemianopia should be excluded or, if included, they should be analyzed separately. Finally, the assessment procedures should include both standardized batteries for the evaluation of hemispatial neglect severity, such as BIT, and the evaluation of disability.

## Conclusion

To conclude, the results of the present review show that EP is a promising procedure in the treatment of hemispatial neglect after stroke and that further research in the evaluation of EP is needed.

## Conflict of Interest Statement

The authors declare that the research was conducted in the absence of any commercial or financial relationships that could be construed as a potential conflict of interest.
